# Alcohol and Cigarette Use among Adolescents and Young Adults in Austria from 2004–2020: Patterns of Change and Associations with Socioeconomic Variables

**DOI:** 10.3390/ijerph182413080

**Published:** 2021-12-11

**Authors:** Julian Strizek, Alfred Uhl, Michael Schaub, Doris Malischnig

**Affiliations:** 1 Gesundheit Österreich GmbH (Austrian Public Health Institute), 1010 Vienna, Austria; alfred.uhl@sfu.ac.at; 2 Faculty of Psychotherapy Science, Sigmund Freud University Vienna, 1020 Vienna, Austria; 3 Swiss Research Institute of Public Health and Addiction (ISGF), University of Zürich, 8006 Zurich, Switzerland; michael.schaub@isgf.uzh.ch; 4 Institute for Addiction Prevention, Office of Addiction and Drug Policy of Vienna, 1030 Vienna, Austria

**Keywords:** alcohol consumption, smoking, young adults, adolescents, survey data, trend analyses

## Abstract

Background: Adolescents and young adults are a crucial target group for preventing harm related to substance use. Recently, declining alcohol and tobacco consumption in young people has been observed in many countries. Based on survey data from 2004 to 2020, we describe time trends for several subgroups of adolescents and young adults (based on consumption levels and socioeconomic variables) and analyze associations between the level of alcohol per capita consumption or daily smoking and socioeconomic variables. Methods: Time trends for males and females are analyzed by a two-way ANOVA and predictors of use by using multivariate regression and logistic regression. Results: Alcohol per capita consumption decreased significantly for both sexes in the 16-year period, with male and female consumption levels converging. Daily smoking was equally prevalent for young males and females and decreased to a similar degree for both sexes. Being male and living in rural areas are associated with a higher level of alcohol consumption. Daily smoking is associated with a low level of education and is more prevalent among young adults who have already started to work. Conclusions: The decline in alcohol use and daily smoking among adolescents and young adults is taking place simultaneously. However, higher levels of alcohol consumption and daily smoking occur in different groups of adolescents and young adults, which should be considered in prevention strategies.

## 1. Introduction

Alcohol use has been found to be causally linked to many diseases and injury categories [1]. For those aged from 25–49 years, alcohol use is the leading risk factor for disability-adjusted life years (DALY), representing years lost due to disability, illness, and early death. Tobacco use is the second highest risk factor on a global level and was the leading cause of death in many high-income countries in 2019 [2]. Alcohol and tobacco consumption, therefore, ranks high among public health priorities for young adolescents in affluent countries. At the same time, efforts are being made to improve skills and coping strategies in the general population regarding legal and illegal substances and/or addictive behaviors. Universal prevention is complemented by selective prevention for risk groups and by indicated prevention for those who already show symptoms of addiction (e.g., to prevent relapse) [3]. In Austria, regional addiction prevention institutes organize and offer preventive measures such as life-skill and risk-skill programs (e.g., Gemeinsam stark werden (Growing stronger together), plus, ready4life) for various target groups. The national association “ARGE Suchtvorbeugung” [4] develops guidelines for anyone who is interested in offering preventive measures in the field of addictive behaviors.

With regard to alcohol consumption, the European School Survey Project on Alcohol and other Drugs (ESPAD) revealed a constant downward trend between 1995 and 2015 for the majority of participating countries. In the latest survey from 2019, this decline leveled off in some countries but continued in others, including Austria [5]. Data from the Health Behaviour in School-Aged Children study (HBSC) also indicate that there has been a clear decline, especially in countries with previously high levels of alcohol consumption by adolescents [6,7].

The available evidence is not robust enough to permit firm conclusions as to the reasons for that decline [8,9]. There is evidence that it is affecting all strata of society, while concerns have also been raised that some disadvantaged subgroups are not following this trend. The decline in alcohol consumption seems to be limited to adolescents (aged 18 and under), whereas young adults and adults in many regions did not reduce their consumption [10]. This is exactly what can be observed in Austria, where student data indicate a sharp decline since 2003 [11], although data from the alcohol industry only suggest a minor change in overall per capita consumption over the last two decades [12]. In line with this decline in alcohol consumption in the youngest age groups, some authors argue that it is due to a prolongation of childhood or to a delay in adolescence, which does not only affect substance use but also affects other behaviors related to late adolescence, like having a first job [13]. In most countries, the decline in alcohol consumption is particularly strong among young males. As a consequence, some authors suggest that alcohol has lost its role in youth culture as a rite of passage ritual or as an identity-forming element of (predominantly) male adolescence [14,15]. In this interpretation, the sober life style among young people seems to reflect a common understanding of legitimate behavior: being moderate, nuanced, and in control [16]. For this reason, for a project to be successful, it has proven essential for the peers of the target group concerned to be involved in the development of preventive measures.

Alcohol consumption is not distributed equally among young people. There is some evidence that young people in rural areas drink more than those in urban areas [17,18] and report binge drinking more frequently [19]. Increased rates for alcohol use are thought to be more common in rural areas due to more permissive attitudes toward legal substances [20] or a lack of leisure time activities not related to substance use [21]. In contrast, the prevalence rates for using illicit substances are thought to be higher in urban areas [22] due to easier access to illicit substances in urban areas. However, differences between rural and urban areas in terms of illicit drug use seem to be narrowing or even vanishing entirely [19].

Changes in drinking behavior at the population level can occur collectively (i.e., they apply to all levels of consumers in a similar manner as postulated by Skog [23]) or they may accentuate differences between low-level and high-level drinkers (i.e., a polarization of drinking habits as posited by Hallgren et al. [24]. A recent study [25] based on student data indicates that in 15 European countries the decline in alcohol consumption happened collectively, whereas in 11 countries a polarization of drinking patterns occurred (more individuals consuming less and more individuals consuming more).

Similar to alcohol consumption, tobacco smoking is on the decline in many European countries [26]. In contrast to alcohol, however, the reduction in tobacco consumption goes hand in hand with more restrictions on the availability and affordability of tobacco products. Consequently, this trend is less surprising and is often attributed to a tighter tobacco control policy [27,28]. Even though this interpretation is tempting in the tobacco field, we should keep in mind that such studies are based on ecological correlations (i.e., policy measures and prevalence rates at the population level) and, therefore, caution is warranted when results are extrapolated to individual behavior. In Austria, for example, smoking among adolescents decreased even before tighter tobacco control measures were introduced [29]. Surprisingly, analyses based on HBSC data suggest that pricing policies do affect the smoking habits of male adolescents but not of females [30,31]. The decline in the consumption of traditional cigarettes is also accompanied by an increase in alternative ways to consume nicotine (e.g., e-cigarettes, smoke-free tobacco), which are particularly popular among younger consumers of nicotine products [5,32]. Data from the UK did not support concerns about a ‘renormalisation’ of smoking as a consequence of increased experience with e-cigarettes among adolescents [33].

Smoking has a strong social gradient. Disadvantaged groups are more likely to start smoking, and their attempts to quit smoking are less successful [34,35]. Results from the latest Austrian Health Interview Survey (ATHIS) indicate a particularly strong social gradient for daily adult smokers: respondents with a lower educational level are twice as likely to be daily smokers as respondents with the highest level of education [36]. Students with a low socioeconomic status also showed an increased risk for daily smoking in a Danish sample of HBSC students [37]. Apart from socioeconomic differences, there is also evidence for a strong demand to adjust smoking cessation programs to the cultural background of smokers [38].

In our paper, we provide detailed descriptive information on trends for both alcohol and cigarette consumption among adolescents and young adults in Austria to highlight the similarities and differences in the developments for both substances. Our analyses are based on four waves of general population surveys (2004, 2008, 2015, 2020) covering a time period of 16 years [39,40,41]. The trends are analyzed for different subgroups (sex, educational level, employment status, place of residence) to assess whether the decline occurred homogenously across all adolescents and young adults or whether some subgroups were affected more strongly than others. In addition, we assess changes in different levels of consumption to detect polarization or collective shifts. Finally, we try to identify variables associated with substance use using a pooled data set of all survey waves and controlling for time trends.

## 2. Materials and Methods

### 2.1. Data Sources

The data were provided by four national, representative samples of the Austrian resident adult population (15 years and older) in projects commissioned and funded by the Austrian Ministry of Health. The surveys were conducted following the guidelines for general population surveys issued by international bodies like the EMCDDA [42] or recommendations from international expert groups in alcohol epidemiology [43]. Participation in all four surveys was completely voluntary and anonymous.

Homeless people, people living in institutions, and people with severe learning deficits were not included in our samples. Data collection was conducted face-to-face in 2004 and 2008, using a split-half method (face-t-face and online) in 2015, and completely online in 2020. In both 2015 and 2020, the online interviewees were selected from a predefined panel representative of the Austrian population. Analysis of the 2015 data did not show any relevant effect of the mode of collection on the observed prevalence rates [41].

### 2.2. Target Group and Weighting

For all analyses presented in this paper, only data from respondents aged between 15 and 24 were considered. In all four survey waves, younger age groups were oversampled, resulting in a disproportionately larger sample for those age groups (2004: *n* = 989, 2008: *n* = 1824, 2015: *n* = 829; 2020: *n* = 1126). The data from different survey waves were weighted based on the overall distribution of age and sex to control for potential effects of those two variables on the overall trend of prevalence rates. Analyses including information on their educational level and employment status were restricted to young adults aged between 20 and 24. Below, the term “young adults” will always refer to 20- to 24-year-olds, with the term “adolescents and young adults” referring to 15- to 24-year-olds.

### 2.3. Measures

Alcohol consumption and cigarette use were analyzed in time trends and used as dependent variables in multivariate analyses.

The concept of a ‘standard drink’ reveals great variation in different drinking cultures [44] and, therefore, the results of alcohol per capita consumption are indicated in grams of alcohol a day (g/d). Twenty grams of alcohol translate to half a liter of beer of normal strength or a quarter of a liter of light wine. Grams of alcohol per day were calculated following the recommendations of the Standardized European Alcohol Survey [43] using a beverage-specific quantity–frequency measure.

Cigarette consumption was assessed in terms of the percentage of daily/almost daily smokers and transformed into a dichotomous variable. In addition, the number of cigarettes smoked per day was used as a measure to indicate the intensity of smoking behavior.

Sex (male/female), educational level, employment status, and place of residence were used as dichotomous indicator variables for both alcohol per capita consumption and daily smoking. Educational level distinguishes between a low-level (corresponding to ISCED level 3 or lower) and a high-level (corresponding to ISCED level 4 or higher). Employment status distinguishes between “working” and “in education”. Unemployed respondents and those on parental leave were assigned to the category “other”. As there turned out to be very few cases in this last category, it was dropped for further analysis so as to avoid random variations due to confounders unrelated to employment status. Place of residence distinguishes between rural (living in the countryside or small villages) and urban settings (living in towns or metropolitan areas) based on the main residence of the respondent.

### 2.4. Analyses

The time trends in Section 3.1 provide descriptive information on changes in mean per capita consumption (including abstention) and in the prevalence of daily smokers. The overall change for males and females is displayed in charts and change rates were calculated for different subgroups. A two-way ANOVA was used to assess significant differences (alpha level = 0.05) between the two sexes, survey years, and the interaction term (sex * survey year). A significant effect of the interaction term indicates that the association between sex and the dependent variable differs between survey years. Interactions terms were also calculated to assess temporal changes in the association between educational level, employment status, place of residence, and the dependent variable.

In Section 3.2, trends are displayed for different levels of consumption using quartiles of alcohol per capita consumption and quartiles for the number of cigarettes smoked a day. Data from respondents who abstain from alcohol or do not smoke at all were excluded from these analyses.

In Section 3.3, multivariate analyses were carried out based on the pooled sample of the four survey waves to predict alcohol per capita consumption and daily smoking. Trend effects were controlled for by using a dichotomous indicator variable for each survey year. Sex, educational level, employment status, and place of residence were included as dependent variables in the final model. For the metric outcome of alcohol per capita consumption, multiple regression was applied. For the dichotomous outcome of daily smoking, binary logistic regression was applied. Significance was assessed using an alpha level of 0.05. All analyses were performed using SPSS 24 (IBM, Amonk, NY, USA).

## 3. Results

### 3.1. Changes in Alcohol and Nicotine Consumption between 2004 and 2020

**Figure 1:**
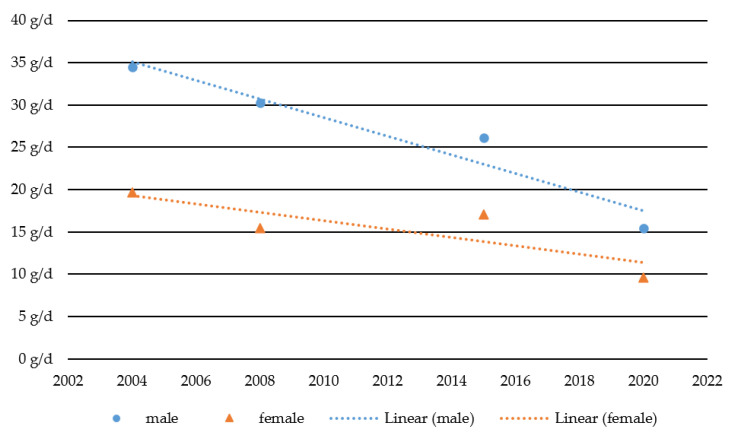
Change in alcohol per capita consumption for male and female adolescents and young adults between 2004 and 2020. Note: g/d = gram/day.

Figure 1 indicates that alcohol per capita consumption among adolescents and young adults in Austria declined by more than 50% (from 27 g/d to 13 g/d) between 2004 and 2020. The decline in per capita consumption among males (from 34 g/d to 15 g/d or −54%) is slightly larger than among females (from 20 g/d to 10 g/d or −51%). Consequently, the gap between males and females narrowed, both in absolute and relative terms. In 2004, males consumed 15 g more alcohol a day on average, which is 75% more than females. By 2020 this divergence between the two sexes had decreased to 6 g a day, with males consuming 60% more than females on average. A two-way ANOVA revealed significant effects for sex (*p* = 0.000) and survey year (*p* = 0.000) as independent variables as well as for the sex * survey year interaction term (*p* = 0.003).

Based on educational level, employment status, and place of residence, all subgroups showed a downward trend in alcohol consumption (Table 1). Young adults with a higher level of education exhibited a steeper decrease than those with a lower level (−60% vs. −45%), as did young adults still in education compared to those who were already working (−62% vs. −51%). Adolescents and young adults in urban settings showed a larger decline compared to adolescents and young adults in rural settings (−53% vs. −47%). Effect sizes for the interaction terms are rather small and, therefore, indicate only very small changes in the association between alcohol per capita consumption and the three selected socioeconomic variables. In fact, the association was only significant for employment status.

**Figure 2:**
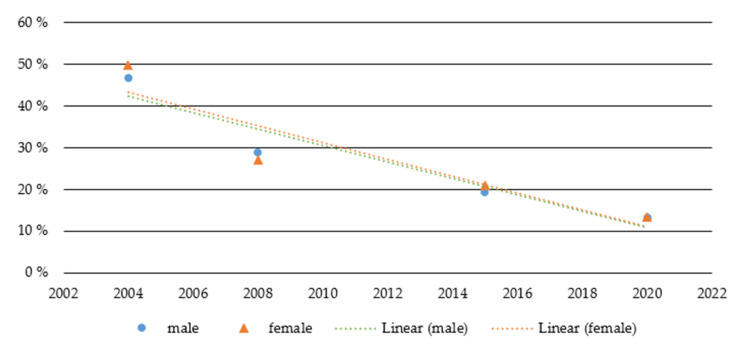
Daily smoking among adolescents and young adults between 2004 and 2020.

In contrast to alcohol use, daily smoking of cigarettes started at similar base levels for males and females and decreased for both sexes to an almost identical degree. Overall, the number of daily smokers among adolescents and young adults declined from 48% to 13% (males from 47% to 13%, −73%; females from 50% to 13%, −72%). A two-way ANOVA revealed a significant effect for survey year (*p* = 0.000) but no significant effects for sex (*p* = 0.638) or the interaction term sex * survey year (*p* = 0.516) (Figure 2).

Based on educational level, employment status, or place of residence, all subgroups showed a decrease in the prevalence of daily smoking. Young adults with a higher level of education showed a steeper decrease (−73%) than those with a lower educational level (−54%) with a similar pattern for young adults still in education (−76%) compared to those already in work (−67%). Adolescents and young adults in rural settings (−77%) exhibited a larger decline compared to those in urban settings (−70%). Effect sizes for the interaction terms are rather small and, therefore, indicate only very small changes in the association between daily smoking and the three selected socioeconomic variables. Again, the association was only significant for employment status (Table 2).

### 3.2. Trends for Different Levels of Consumption

**Figure 3:**
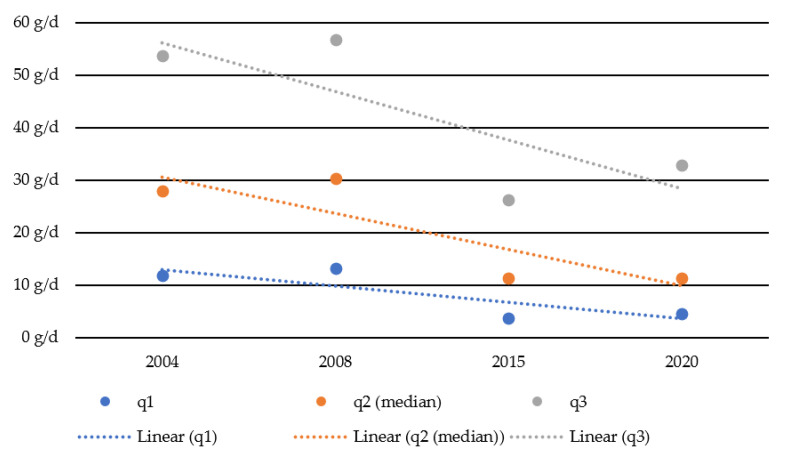
Quartiles of alcohol per capita consumption (g/d) among the drinking population from 2004–2020.

The quartiles of consumption levels among the drinking population of adolescents and young adults are displayed in Figure 3. Compared to 2004, the value for the upper quartile (q3) dropped from 54 g/d to 32 g/d (−39%), the value for the median (q2) fell from 28 g/d to 11 g/d (−59%), and the lowest quartile (q1) dropped from 12 g/d to 5 g/d (−62%). Accordingly, the gap between high- and low-level drinkers narrowed in absolute terms from 42 g in 2004 to 28 g in 2020. The decrease in alcohol consumption can be observed at all levels of consumption.

**Figure 4:**
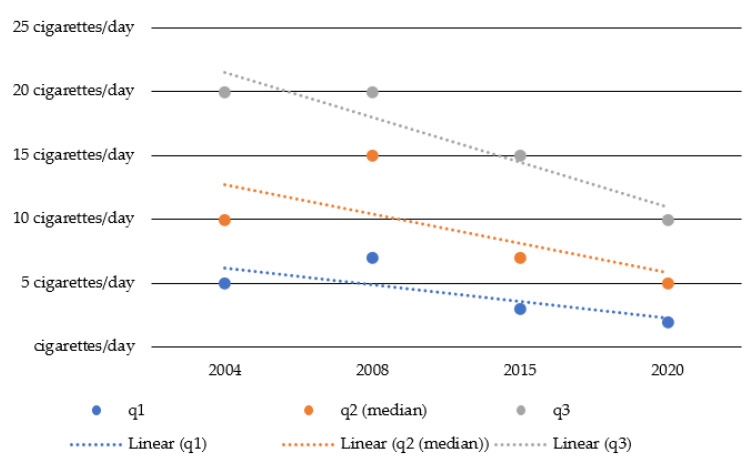
Quartiles of cigarettes a day among the smoking population between 2004 and 2020.

The quartiles of the number of cigarettes a day among the smoking population of adolescents and young adults are illustrated in Figure 4. Compared to 2004, the value for the upper quartile (q3) dropped from 20 to 10 cigarettes (a reduction of 50%), the value for the median (q2) dropped from 10 to 5 cigarettes (a reduction of 50%), and the lower quartile (q1) dropped from 5 to 2 cigarettes (a reduction of 60%).

Similar to alcohol consumption, the gap between high- and low-level smokers narrowed in terms of absolute numbers (from 15 cigarettes difference in 2004 to 8 cigarettes difference in 2020). The decrease in tobacco consumption can be observed at all levels of consumption as well.

### 3.3. Associations between Socieconomic Variables and Alcohol per Capita Consumption or Daily Smoking

In a final step, two multivariate models were set up to assess whether sex, educational level, employment status, or place of residence are significantly associated with alcohol per capita consumption or daily smoking.

Survey years were used as indicator variables in Model 1, with sex, educational level, employment status, and place of residence used as additional variables in Model 2. Adding the four additional variables increases the predictive values (*R*^2^) for the level of alcohol per capita consumption and daily smoking (Table 3).

Sex and place of residence significantly predict the level of alcohol per capita consumption. Being male and living in rural areas is associated with a higher level of alcohol consumption in the pooled sample of young adults (20–24 years). In contrast, the level of education and employment status do not significantly predict alcohol per capita consumption (Table 4).

According to the logistic regression model, young adults with a higher level of education and young adults who are still in education are less likely to smoke on a daily basis. In contrast, sex and place of residence do not predict whether young adults smoke cigarettes on a daily basis or not (Table 5).

## 4. Discussion

While most studies focus either on alcohol or tobacco consumption, our analyses help to highlight the similarities and differences between the two substances based on the same data set covering a period of 16 years. Our results provide empirical evidence that the decline in both alcohol and tobacco consumption among adolescents and young adults is also reflected in the latest survey data. These results are in line with most results from the ESPAD study [5], according to which Austria belongs to the group of countries where alcohol use has declined to date. Using broader age groups than in the school surveys, our results support the idea that the decrease in drinking and smoking is not limited to student populations but is also passed on to subsequent age cohorts.

Both males and females show a downward trend for alcohol and tobacco consumption, but the trends differ in detail: whereas daily smoking for both sexes started at the same level and dropped to a similar extent for both sexes, male alcohol consumption started at a higher level but showed a steeper decline than female alcohol consumption. Despite the convergence of drinking levels between men and women [45], being male is still associated with higher levels of alcohol per capita consumption. Furthermore, a qualitative study on adolescents who abstain indicate that gender roles are more flexible but that males and females still face different normative expectations with regard to drinking behaviors [15]. In contrast, levels of cigarette smoking do not show any differences between males and females at all. These results are in contrast to international statistics provided by the WHO which indicate a much higher rate of smoking in males than in females both for Europe and on a global level [26].

Based on the different levels of consumption for the respondents in our sample, our analyses do not fuel concerns that the general decline in legal substance use may lead to a polarization of consumption cultures (meaning that the decrease can be explained only by a growing number of abstainers, with active consumers not showing any reduction). Instead, both high- and low-level consumers of alcohol and tobacco show a reduction, resulting in a convergence of consumption patterns. This result is important and requires further observation with regard to recent concerns that in the course of the COVID-19 pandemic, health disparities in general, as well as health problems in relation to problems due to substance use in particular, may have grown [46,47]. In this context, our subgroup analyses based on data before the pandemic revealed consistent trends for educational level and employment status over the period of 16 years: declines are observed in all subgroups but are more pronounced among young adults still in education. Therefore, further research is required to ascertain whether this trend will continue after the pandemic and whether the social gradient of substance-related problems will steepen. Holstein et al. [37] stress in this context that studies of changes in socioeconomic differences over time should address both absolute and relative socioeconomic differences as they may lead to different conclusions.

Despite the similarities in decreasing consumption, alcohol and cigarette use differs in terms of their associations with socioeconomic variables. Sex and place of residence are significantly associated with the level of alcohol consumption, whereas socioeconomic status (educational level, employment status) does not show any significant effect on per capita consumption. Exactly the opposite can be observed for cigarette use. Educational levels and employment status are significantly associated with daily smoking, whereas the prevalence rates for daily smoking do not show any significant difference between the two sexes or between rural and urban areas.

## 5. Limitations

Several limitations should be considered when interpreting the results presented in this paper. First, our results are based on self-reported data. Changes in attitudes toward alcohol or tobacco consumption may influence the likelihood of truly reporting the magnitude of consumption, although this effect is expected to be smaller compared to survey research on illegal substance use. Second, any conclusion as to a polarization of consumption patterns is limited by the fact that marginalized people are under-represented or even excluded (e.g., homeless people) in surveys. Third, our analyses were limited to indicators covered in all survey waves. For example, there is growing evidence [1] that the detrimental health effects of alcohol consumption are not only caused by the amount of per capita consumption but also by patterns of alcohol consumption (e.g., the likelihood of injury increases when large amounts of alcohol are consumed in a short time). However, patterns of consumption were not assessed in all survey waves and, therefore, could not be included in our analyses. Finally, the data collection in 2020 coincided with the first wave of the COVID-19 pandemic in Austria. The vast majority of respondents reported that they had not notably changed their alcohol or cigarette consumption due to the pandemic. Still, it is not possible to separate effects specific to circumstances caused by the pandemic from the continued decline in consumption.

## 6. Conclusions

The convergence of male and female alcohol consumption levels among young adults is one of the most relevant changes in substance use in the 16-year period. Differences in the prevalence of smoking between males and females already vanished two decades ago. However, similar levels of consumption should not hide the fact that more specific prevention measures might be required for both sexes separately and for different substances. The associations with socioeconomic variables are rather stable over the 16-year period, and preventive measures should consider that different subgroups of adolescents and young adults show higher levels of alcohol use compared to tobacco consumption. Regarding alcohol use, specific interventions for young males living in rural areas might be necessary to complement existing interventions. Regarding smoking, more targeted interventions may be required for young adults with lower levels of education and who have already started to work. Our data do not provide evidence for a polarization of drinking or smoking patterns. However, this issue should not be neglected due to the potential consequences of the COVID-19 pandemic on the social gradient of substance use.

## Figures and Tables

**Table 1:** Change in mean per capita consumption (g/d) for different subgroups of adolescents and young adults between 2004 and 2020.

Subgroup	2004	2008	2015	2020	Change Rate 2004–2020 ^1^	Interaction Term (Eta Square)
Total	27	23	22	13	−54%	-
Sex						
Males	34	30	26	15	−55%	0.003 **
Females	20	15	17	10	−51%	
Educational level *						
Lower level of education	32	23	28	17	−45%	0.001
Higher level of education	34	23	22	14	−60%	
Employment status *						
Working	34	23	28	16	−51%	0.003 **
In education	35	30	22	13	−62%	
Place of residence						
Rural	29	24	28	16	−47%	0.001
Urban	26	21	19	12	−53%	

^1^ formula used for change rate: 1—mean^2020^/mean^2004^; * based on respondents aged 20–24; ** significant.

**Table 2:** Prevalence of daily smoking in different subgroups of adolescents and young adults between 2004 and 2020.

Subgroup	2004	2008	2015	2020	Change Rate 2004–2020 ^1^	Interaction Term (Eta Square)
Total	48.2%	28.0%	20.1%	13.3%	−72.5%	
Sex						
Male	46.8%	28.9%	19.3%	13.3%	−71.6%	0.000
Female	49.7%	27.1%	20.9%	13.3%	−73.2%	
Educational level *						
Lower level of education	64.5%	38.0%	37.0%	29.6%	−54.1%	0.001
Higher level of education	39.6%	21.6%	15.5%	10.6%	−73.2%	
Employment status *						
Working	57.1%	32.9%	26.5%	18.9%	−67.0%	0.005 **
In education	31.4%	14.1%	17.7%	7.4%	−76.3%	
Place of residence						
Rural	50.8%	27.8%	21.5%	11.7%	−77.1%	0.001
Urban	46.3%	28.2%	19.2%	13.8%	−70.3%	

^1^ formula used for change rate: 1 − mean^2020^/mean^2004^; * based on respondents aged 20–24; ** significant.

**Table 3:** Model summaries.

Independent Variable	Model	*R*	*R* ^2^	Change in *R*^2^	Change in *F*	Sig. Change in *F*
Alcohol per capita consumption	Model 1	0.151	0.021	0.023	17.989	0.000
	Model 2	0.239	0.054	0.034	21.153	0.000
		−2 Log-Likelihood	Cox & Snell *R*^2^	Nagelkerkes *R*^2^		
Daily smoking	Model 1	2653.5	0.064	0.091		
	Model 2	2528.0	0.113	0.161		

**Table 4:** Regression parameters for predictors of per capita consumption (young adults between 2004 and 2020).

Model	Predictors	*B*	Stand. Error	Beta	*T*	sig.
Model 1	Constant	34.14	1.84		18.52	0.00
	Survey year 2008	−9.84	2.30	−0.12	−4.27	0.00
	Survey year 2015	−8.67	2.70	−0.08	−3.22	0.00
	Survey year 2020	−18.84	2.57	−0.19	−7.33	0.00
Model 2	Constant	61.83	4.90		12.61	0.00
	Survey year 2008	−10.34	2.29	−0.12	−4.52	0.00
	Survey year 2015	−8.17	2.66	−0.08	−3.08	0.00
	Survey year 2020	−17.55	2.56	−0.18	−6.87	0.00
	Sex	−14.86	1.69	−0.18	−8.81	0.00
	Educational level	−1.13	1.92	−0.01	−0.59	0.56
	Employment status	1.45	1.99	0.02	0.73	0.46
	Place of residence	−3.80	1.75	−0.05	−2.17	0.03

**Table 5:** Regression parameters for predictors of daily smoking (young adults between 2004 and 2020).

Model	Predictors	*B*	Stand. Error	Wald	*df*	sig.	exp(B)
Model 1	Constant	3.71	0.26	211.6	1	0.00	40.84
	Survey year 2008	−0.77	0.12	44.6	1	0.00	0.46
	Survey year 2015	−1.14	0.14	62.2	1	0.00	0.32
	Survey year 2020	−1.71	0.15	125.7	1	0.00	0.18
Model 2	Constant	4.53	0.29	238.8	1	0.00	92.54
	Survey year 2008	−0.99	0.12	64.7	1	0.00	0.37
	Survey year 2015	−1.15	0.15	59.1	1	0.00	0.32
	Survey year 2020	−1.65	0.16	109.5	1	0.00	0.19
	Sex	0.08	0.10	0.7	1	0.39	1.09
	Educational level	−0.79	0.11	54.0	1	0.00	0.45
	Employment status	−0.64	0.13	25.2	1	0.00	0.53
	Place of residence	0.04	0.10	0.2	1	0.68	1.04

## Data Availability

All data and SPSS syntax used for the analyses in this paper can be made available upon request.
